# West Nile Virus: Peterson et al. Respond

**Published:** 2006-09

**Authors:** Robert K.D. Peterson, Paula A. Macedo, Ryan S. Davis

**Affiliations:** Montana State University Bozeman, Montana E-mail: bpeterson@montana.edu

We thank Schofield et al. for their interest in our article and for their comments. We would like to clarify that Peterson et al. (2006) is simply a screening-level (tier 1) risk assessment in which we separately and conservatively examined the residential human risks from exposure to West Nile virus (WNV) and mosquito adulticides. As with all screening-level risk assessments, our assessments were not refined, but they did reveal the magnitude of risk compared to relevant end points. As Schofield et al. point out, our article should not be misinterpreted to indicate that the health risks associated with adulticiding are offset by its potential for WNV reduction. This is because we did not conduct a risk–benefit assessment, which was beyond the scope of our study.

Our article (Peterson et al. 2006) represents an initial step in an ongoing multiyear analysis of risk issues associated with certain vectorborne diseases and vector management strategies. We plan to address some of the issues Schofield et al. raise in subsequent papers.
